# Risk of bowel ischemia in patients with mesenteric neuroendocrine tumors after treatment with ^177^Lu-DOTATATE

**DOI:** 10.1530/EO-25-0033

**Published:** 2025-07-07

**Authors:** Eleonora Pelle, Taymeyah Al-Toubah, Ghassan El-Haddad, Brian Morse, Bhavana Konda, Vineeth Sukrithan, Jonathan Strosberg

**Affiliations:** ^1^ Department of Gastrointestinal Oncology, H Lee Moffitt Cancer Center and Research Institute, Tampa, Florida, USA; ^2^ Department of Diagnostic Imaging and Interventional Radiology, H Lee Moffitt Cancer Center and Research Institute, Tampa, Florida, USA; ^3^ Division of Medical Oncology, Department of Internal Medicine, Ohio State University College of Medicine, Columbus, Ohio, USA

**Keywords:** PRRT, bowel ischemia, small bowel NET, mesenteric lesions

## Abstract

**Background:**

Lutetium-177 (^177^Lu)-DOTATATE is an effective treatment for metastatic gastroenteropancreatic (GEP) NETs. However, radiation can cause transient inflammation/swelling of tumors, which can result in toxicity. Treatment-related small bowel obstruction associated with mesenteric or peritoneal disease has been described. We investigated the potential for intestinal ischemia in ^177^Lu-DOTATATE-treated patients.

**Methods:**

Clinical records were reviewed of patients with midgut NETs treated with ^177^Lu-DOTATATE at the Moffitt Cancer Center between April 2018 and December 2022 and at The Ohio State University between December 2017 and October 2020.

**Results:**

Among the cases reviewed, we identified three patients who developed bowel ischemia/perforation shortly after their initial treatment with ^177^Lu-DOTATATE. All patients had metastatic small bowel NET with prominent mesenteric mass encasing/obstructing the mesenteric vessels and preexisting symptoms of postprandial abdominal pain.

**Conclusion:**

Acute bowel ischemia may be a rare complication of PRRT in patients with mesenteric arterial or venous obstruction from mesenteric metastasis.

## Introduction

Well-differentiated midgut (small bowel) neuroendocrine tumors (NETs) are characterized by a high propensity to metastasize to mesenteric lymph nodes, peritoneum, and liver. Mesenteric metastases often exhibit desmoplastic features, possibly related to the local secretion of serotonin (Druce *et al.* 2009, Cives & Strosberg 2018). When located at the root of the mesentery, tumors are usually unresectable and can occlude the superior mesenteric artery (SMA) and/or superior mesenteric vein (SMV), potentially resulting in bowel ischemia (Pelle *et al.* 2021).

Lutetium-177 (^177^Lu)-DOTATATE is a radiolabeled somatostatin analog (SSA) approved for treating patients with metastatic gastroenteropancreatic (GEP) NETs after progression on SSA therapy. The NETTER-1 phase III trial demonstrated that ^177^Lu-DOTATATE significantly improved progression-free survival compared to high-dose octreotide in patients with advanced midgut NETs. Side effects observed in the trial consisted primarily of myelotoxicity and acute nausea, the latter mainly attributable to the amino acid infusions used to prevent nephrotoxicity (Strosberg *et al.* 2017).

Several small case series have recently described a risk of bowel obstruction related to PRRT in patients with midgut NETs who have a significant burden of peritoneal or mesenteric disease. The mechanism of obstruction appears to be related to an inflammatory response elicited by the delivery of radioactivity to intra-abdominal tumors. Radiation mesenteritis or peritonitis can cause tumors to adhere to adjacent intestines, resulting in intestinal blockage and even frozen abdomen in severe cases (Merola *et al.* 2020, Blažević *et al.* 2021, Strosberg *et al.* 2021, Al Mansour *et al.* 2024).

Another distinct intestinal complication of radiolabeled SSA therapy may be related to bowel ischemia. The same inflammatory response to radiation can also exacerbate preexisting occlusion of mesenteric arteries and/or veins, leading to intestinal angina and even infarction/perforation. In this case series, we describe intestinal ischemia events that appear to have been exacerbated by treatment with ^177^Lu-DOTATATE.

## Methods

A database of patients at the Moffitt Cancer Center (MCC) with GEP-NETs who received ^177^Lu-DOTATATE treatment between April 2018 and December 2022 was reviewed to identify cases of intestinal ischemia leading to hospitalization during or shortly after PRRT. Institutional review board approval was obtained with a waiver of consent due to the study’s retrospective nature.

Separately, a review of all patients with neuroendocrine tumors who received ^177^Lu-DOTATATE between December 2017 and October 2020 at The Ohio State University was conducted. Approval was obtained from the Institutional Review Board of The Ohio State University (OSU), and permission was obtained for a waiver of consent.

## Results

### MCC cohort

291 patients with GEP-NETs were treated with ^177^Lu-DOTATATE between 4/2018 and 12/2022. Among these patients, 121 had mesenteric metastases present at the time of treatment. Two patients (1.6% of patients with mesenteric disease) were identified who developed complications of bowel ischemia of sufficient severity to result in hospitalization.

### OSU cohort

A total of 104 patients treated with ^177^Lu-DOTATATE were identified, of which 91 were GEP-NETs (Sukrithan *et al.* 2024). Of these, 28 had mesenteric metastases present at the time of treatment. One patient (3.6% of patients with mesenteric disease) developed bowel ischemia leading to death.

**Figure 1:**
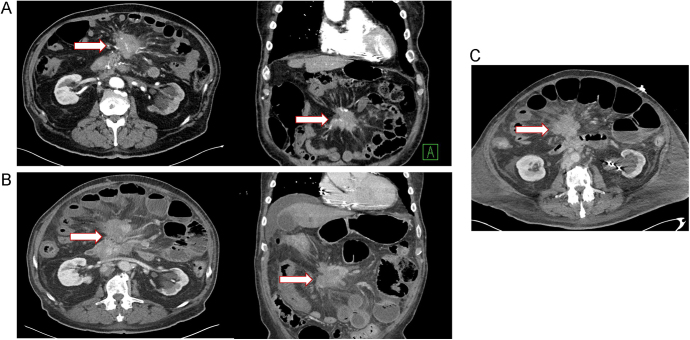
CT scan of patient 1. (A) A mesenteric mass with confluent lymphadenopathy causing encasement and occlusion of the SMV, and severe narrowing of the SMA. (B) CT scan performed after the initial cycle of ^177^Lu-DOTATATE showing interval dilation of the small bowel with some air-fluid levels, and wall thickening of the mid to distal small bowel with pneumatosis, adjacent small foci of free air, and gas within mesenteric veins, likely related to bowel ischemia. (C) Diffuse dilation of the small bowel and pneumatosis intestinalis consistent with ischemic changes, and no focal evidence of obstruction.

The cases are described below:

**Case 1:** A 75-year-old man had been diagnosed with metastatic grade 1 small bowel NET and carcinoid syndrome 11 years before initiating ^177^Lu-DOTATATE treatment. Other than somatostatin analog (SSA) therapy, his prior treatments included radiation to a retroperitoneal lymph node and vertebral metastasis. His disease was metastatic primarily to mesenteric and retroperitoneal LNs and bone. Before beginning ^177^Lu-DOTATATE, he complained of postprandial abdominal pain and weight loss. Pre-treatment CT described a mesenteric mass with confluent lymphadenopathy measuring approximately 6 cm in diameter (Fig. 1A), causing encasement and occlusion of the SMV and severe narrowing of the SMA.

Nine days after the initial cycle of ^177^Lu-DOTATATE, the patient developed severe exacerbation of abdominal pain. He was admitted to the hospital where repeat CT revealed interval dilation of the small bowel with some air-fluid levels and wall thickening of the mid to distal small bowel, with pneumatosis and adjacent small foci of free air as well as gas within mesenteric veins, likely related to bowel ischemia (Fig. 1B). The mesenteric mass occluded the SMV. In addition, there was increased attenuation of the portal vein, potentially due to low venous volume. Repeat CT a day later showed diffuse dilation of the small bowel and pneumatosis intestinalis consistent with ischemic changes, and no focal evidence of obstruction (Fig. 1C).

The patient was treated with bowel rest, nasogastric tube decompression, broad-spectrum antibiotics, and high-dose corticosteroids. Unfortunately, his condition declined, and he expired approximately 3 weeks after admission.

**Figure 2:**
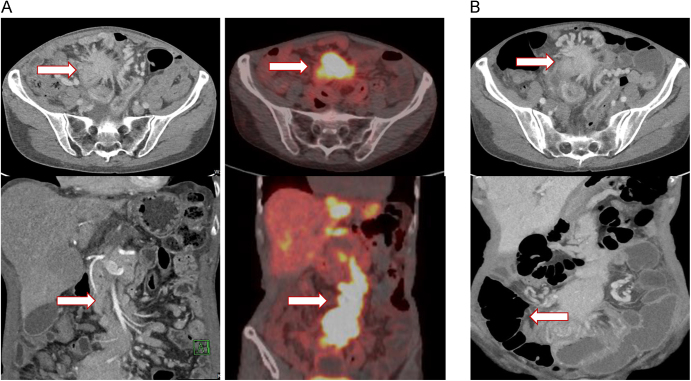
CT and ^68^Ga-DOTATATE PET scan of patient 2. (A) Pre-treatment CT scan and ^68^Ga-DOTATATE PET showing large central mesenteric mass and retroperitoneal lymphadenopathy. (B) Post-treatment CT scan showing a stable 5.6 cm mesenteric mass with chronic SMA encasement and distal occlusion of the SMV.

**Case 2:** A 70-year-old woman had been diagnosed with metastatic grade 2 small bowel NET 3.5 years before initiation of ^177^Lu-DOTATATE treatment. Her initial presentation was in the setting of a bowel obstruction. Laparoscopy showed a matted small bowel associated with a large, unresectable mesenteric mass, and she underwent a surgical small bowel bypass. ^68^Ga-DOTATATE PET showed extensive disease in the liver, large central mesenteric mass and retroperitoneal lymphadenopathy (Fig. 2A). She began treatment with octreotide LAR. Before initiating ^177^Lu-DOTATATE, she developed increased postprandial abdominal pain and nausea. The pre-treatment scan also showed chronic SMV occlusion and dilation of a pelvic loop of bowel due to the mesenteric mass. Due to the enlargement of hepatic metastases, PRRT was recommended. Prophylactic dexamethasone was administered immediately following ^177^Lu-DOTATATE treatment, starting at 4 mg daily and tapering slowly over 3 weeks, in order to minimize the risk of bowel obstruction.

Ten days later, she presented to the hospital with increased abdominal pain. CT scan showed a stable 5.6 cm mesenteric mass with chronic SMA encasement and distal occlusion of the SMV (Fig. 2B). Associated with the mass was a thick-walled hyperemic bowel loop with a 1.3 cm external air-fluid level suggestive of possible contained perforation. There was no obstipation, and oral contrast reached the colon without extravasation. Five days later, her condition worsened acutely, and a CT scan showed a new pneumoperitoneum. She was supported aggressively for a month with parenteral nutrition and broad-spectrum antibiotics, but was eventually transitioned to comfort care, and expired approximately 2 months after admission.

**Figure 3:**
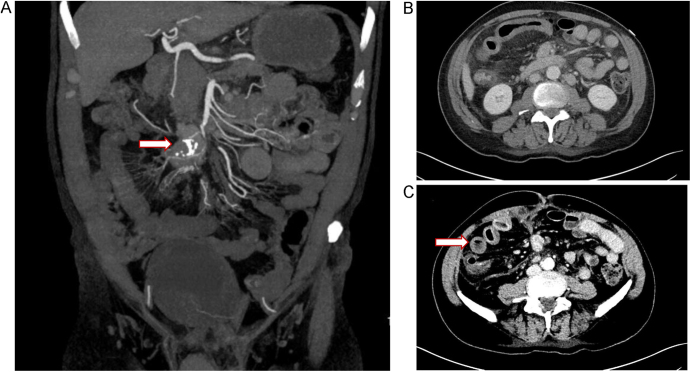
CT scan of patient 3. (A) Mesenteric mass causing stenosis of the distal branches of the SMA. (B) Post-PRRT: long segment of ileum with dilation and circumferential wall thickening. (C) Mucosal hyperenhancement indicating ischemia.

**Case 3:** A 70-year-old male had been diagnosed with metastatic well-differentiated neuroendocrine tumor of unknown grade 8 years before treatment with ^177^Lu-DOTATATE. Before diagnosis, the patient had an 8-year history of intermittent bloating and chronic abdominal pain, which led to imaging that revealed a mesenteric mass and liver metastases. The patient did not tolerate an initial dose of SSA due to symptoms of worsened diarrhea and abdominal cramping. Due to the unresectable nature of the mesenteric mass, a strategy of watchful waiting was employed, which lasted for 5 years. Subsequently, in the setting of a particularly severe episode of acute-on-chronic abdominal pain, a CT angiogram revealed a calcified mesenteric mass impinging on mesenteric vessels with probable stenosis of the distal branches of the SMA, as well as occlusion of the SMV, bowel wall thickening, and mucosal enhancement of loops of small bowel radiating around the mesenteric mass with some mesenteric congestion (Fig. 3). This led to the initiation of a narcotic (hydromorphone), and 20 mg of octreotide LAR. However, despite escalating doses of narcotics, his abdominal pain worsened over the course of a year. This led to a decision to treat with ^177^Lu-DOTATATE after a risk/benefit discussion. The patient was admitted to the hospital 2 days after the administration of ^177^Lu-DOTATATE for severe abdominal pain and intolerance of oral intake. A CT scan of the abdomen showed a new segment of circumferential small bowel wall thickening with mucosal hyperenhancement and dilation consistent with ischemic enteritis. Total parenteral nutrition was started, and bowel rest was initiated. Subsequently, the patient developed a bowel perforation and died 9 weeks later.

## Discussion

^177^Lu-DOTATATE is generally a well-tolerated therapy for progressive GEP-NETs (Das *et al.* 2019); however, risks of radiation include inflammation and fibrosis. These risks are of particular concern among patients with peritoneal and mesenteric disease. Patients with extensive peritoneal carcinomatosis or mesenteric disease tethering loops of bowel appear to be at high risk of PRRT-associated small intestine obstruction (Merola *et al.* 2020, Blažević *et al.* 2021, Strosberg *et al.* 2021, Al Mansour *et al.* 2024). Intestinal ischemia is a distinct toxicity. Our three-case series indicates that patients with preexisting radiographic and clinical evidence of mesenteric vascular obstruction are at risk of exacerbation of bowel ischemia, leading to infarction and perforation.

The risk of bowel ischemia is difficult to quantify, given the rarity of this event. When these three events occurred, nearly 400 patients with GEP-NET had received ^177^Lu-DOTATATE at these two institutions. It is, therefore, likely that this complication occurs in fewer than 1% of treated patients. It is unclear whether prophylactic corticosteroids can prevent this toxicity. One of the three patients developed acute bowel ischemia despite prophylactic steroids.

It is difficult to conclusively prove that exacerbation of bowel ischemia is directly attributable to ^177^Lu-DOTATATE. All three patients had preexisting radiographic evidence of both arterial and venous obstruction and were at high risk of developing this complication spontaneously. However, the timing of the events (in all cases, less than 10 days after the initial treatment) suggests that ^177^Lu-DOTATATE likely had a role in exacerbating this chronic condition.

Limitations of this study include its retrospective nature, small sample size, and limited frequency of the complication explored. It can also be challenging to distinguish between mechanical bowel obstruction and bowel ischemia, since clinical and radiographic findings can overlap in both conditions and ultimately result in perforation.

In summary, patients with advanced NETs and mesenteric tumors with preexisting signs of vascular obstruction may be at increased risk of intestinal ischemia and infarction after treatment with ^177^Lu-DOTATATE. The benefits of treatment in such patients should be weighed against this risk.

## Declaration of interest

JS: Exelixis, consultant; Boehringer Ingelheim, consultant. TA: Curium, consultant. GE: Bayer HealthCare, consultant; Terumo Medical Corporation, consultant; Cellectar Biosciences, consultant; Canon Medical, consultant; NorthStar Medical Radioisotopes, advisory board; NRG Pallas, consulting fees. The rest of the authors have nothing to disclose.

## Funding

This work did not receive any specific grant from any funding agency in the public, commercial, or not-for-profit sector.
